# Perinatal mental distress in a rural Ethiopian community: a critical examination of psychiatric labels

**DOI:** 10.1186/s12888-020-02646-5

**Published:** 2020-05-12

**Authors:** Jil Molenaar, Charlotte Hanlon, Atalay Alem, Dawit Wondimagegn, Girmay Medhin, Martin Prince, Edward G. J. Stevenson

**Affiliations:** 1 School of Global Health, University of Copenhagen, Øster Farimagsgade 5, Building 9, 1353 Copenhagen, Denmark; 2 Centre for Global Mental Health, Health Service and Population Research Department, Institute of Psychiatry, Psychology and Neuroscience, King’s College London, London, UK; 3 Department of Psychiatry, Addis Ababa University, College of Health Sciences, School of Medicine, WHO Collaborating Centre for Mental Health Research and Capacity Building, 6th Floor, College of Health Sciences Building, Tikur Anbessa Hospital, Addis Ababa, Ethiopia; 4 College of Health Sciences, Centre for Innovative Drug Development and Therapeutic Trials for Africa (CDT-Africa), Addis Ababa University, Addis Ababa, Ethiopia; 5 Addis Ababa University, Aklilu-Lemma Institute of Pathobiology, Sifra Selam, Addis Ababa, Ethiopia; 6 King’s College London, King’s Global Health Institute, Room 1.49, Franklin Wilkins Building, 127, Stamford Street, London, UK; 7 Department of Anthropology, Durham University, Dawson Building, South Road, Durham, DH1 3LE UK

**Keywords:** Postnatal depression, Mental health, Culture, Global mental health, Sub-Saharan Africa, Poverty

## Abstract

**Background:**

Perinatal mental distress poses a heavy burden in low- and middle-income countries (LMICs). This study investigated perceptions and experiences of perinatal mental distress among women in a rural Ethiopian community, in an effort to advance understanding of cross-cultural experiences of perinatal mental distress.

**Methods:**

We employed a sequential explanatory study design. From a population-based cohort study of 1065 perinatal women in the Butajira Health and Demographic Surveillance Site, we purposively selected 22 women according to their scores on a culturally validated assessment of perinatal mental distress (the Self-Reporting Questionnaire). We examined concordance and discordance between qualitative semi-structured interview data (‘emic’ perspective) and the layperson-administered fully-structured questionnaire data (‘etic’ perspective) of perinatal mental distress. We analysed the questionnaire data using summary statistics and we carried out a thematic analysis of the qualitative data.

**Results:**

Most women in this setting recognised the existence of perinatal mental distress states, but did not typically label such distress as a discrete illness. Instead, perinatal mental distress states were mostly seen as non-pathological reactions to difficult circumstances. The dominant explanatory model of perinatal mental distress was as a response to poverty, associated with inadequate food, isolation, and hopelessness. Support from family and friends, both emotional and instrumental support, was regarded as vital in protecting against mental distress. Although some women considered their distress amenable to biomedical solution, many thought medical help-seeking was inappropriate. Integration of perspectives from the questionnaire and semi-structured interviews highlighted the important role of somatic symptoms and nutritional status. It also demonstrated the differential likelihood of endorsement of symptoms when screening tools versus in-depth interviews are used.

**Conclusions:**

This study highlights the importance of the wider social context within which mental health problems are situated, specificially the inseparability of mental health from gender disadvantage, physical health and poverty. This implies that public health prevention strategies, assessments and interventions for perinatal distress should be developed from the bottom-up, taking account of local contexts and explanatory frameworks.

## Background

The term perinatal depression is used to refer to depressive symptoms among pregnant women and mothers up to 12 months postpartum [1]. The prevalence of perinatal depression and the ways in which it is experienced in diverse settings across the world are subjects of active debate. A recent systematic review estimated that 13.1% of women in low- and middle- income countries (LMICs) are affected by perinatal common mental disorders (CMD; mostly depression and anxiety), compared to 11.4% of women living in high-income countries (HICs) [2]. Although prevalence estimates vary considerably between reviews, they are typically higher for LMICs than HICs, both for antenatal and postnatal mental disorders [3, 4]. Depressive disorders in pregnant women and mothers are typically associated with reduced quality of life and functional capacity [5, 6]. Additionally, perinatal depression has been linked to preterm delivery, low birth weight, and decreased maternal sensitivity in the postpartum period [7], which can lead to higher rates of morbidity, undernutrition, and poor cognitive development in infants [8].

The concept of perinatal depression is deeply rooted in Western psychiatric practice and there are concerns that ‘exporting’ the notion to non-Western settings might result in what Kleinman has termed a ‘category fallacy’, i.e. applying a nosological category developed for one culture to members of another culture, where the category may lack relevance [9]. Although some researchers hold that perinatal depression is rare in sub-Saharan Africa due to protective perinatal traditional practices [10], qualitative studies in several sub-Saharan countries have demonstrated that women are perceived to be particularly vulnerable to mental distress during the perinatal period [11–17]. However, such distress is usually not recognised as a discrete illness, and its conceptualisation and expression may differ from that in Western settings.

In an effort to advance understanding of perinatal mental distress and disorder, this study investigated perceptions and experiences of perinatal mental distress states in Ethiopia. The study was nested within the P-MaMiE study (Perinatal Maternal Mental disorder in Ethiopia), a population-based cohort of women in rural Ethiopia. In the P-MaMiE participants, the prevalence of CMD was found to be 12% during pregnancy and 5% two months postnatally using a locally validated version of the Self-Reporting Questionnaire [18]. The aim of this study was to provide a deeper understanding of the experience of perinatal mental distress (‘emic’ perspective; as conceptualised from within a specific culture) and examine concordance with fully-structured questionnaire data of mental distress states (‘etic’ perspective; applying externally-derived criteria) [19]. Additional objectives were to document explanatory models for perinatal mental distress states in this population, and to understand women’s coping strategies and help seeking patterns.

## Methods

### Study design

A sequential explanatory strategy was used, in which layperson-administered fully-structured questionnaire data from the P-MaMiE population-based cohort study was combined with qualitative semi-structured interview data [20].

### Study setting

Data were collected in 2005 and 2006. In the P-MaMiE population-based cohort study, 1065 pregnant women were recruited from a predominantly rural health and demographic surveillance site located in and around Butajira, south central Ethiopia [21]. Livelihoods in the study area are predominantly based on farming of maize and “false banana” (ensete), with khat and chili pepper grown as cash crops. The majority of people in the study area are conversant in Amharic. The predominant religious groups are Islam and Christianity (both Orthodox and Protestant) [21]. Healthcare was made available to study participants by the P-MaMiE study.

### Sample

Women in the P-MaMiE cohort were screened for CMDs using the Self-Reporting Questionnaire 20-item (SRQ-20) during the third trimester of pregnancy and two months postnatally. A total of 22 women were purposively sampled for in-depth interviews based on the SRQ-20 scores (see below) and in order to represent a range of urban/rural, Christian/Muslim, and literate/non-literate individuals. All were married (two in polygamous unions). See Table 1.

**Table 1:** Sociodemographic characteristics of study participants

**Number of participants**	22
**Mean age (minimum, maximum)**	29 years (18–39 years)
**Literate**	5 (23%)
**Education type**	
Formal	6 (27%)
Informal	3 (14%)
None	13 (59%)
**Formal schooling (years)**	
Mean (range)	1.1 (0–8)
**Religion**	
Muslim	15 (68%)
Catholic	1 (5%)
Orthodox Christian	2 (9%)
Protestant Christian	4 (18%)
**Residence**	
Rural	19 (86%)
Urban	3 (14%)

### Fully-structured questionnaire data

The SRQ-20 total score was used to classify each respondent to one of four categories (see Table 2).

**Table 2:** Categories used to classify perinatal mental illness

Label	Definition
Non-case throughout (*n* = 4)	SRQ score < 6 at both pregnancy and postnatal time point
Case throughout (*n* = 9)	SRQ score ≥ 6 at both pregnancy and postnatal time point
Pregnancy case (*n* = 5)	SRQ score ≥ 6 at pregnancy time point, SRQ score < 6 at postnatal time point
New-onset postnatal case (*n* = 4)	SRQ score < 6 at pregnancy time point, SRQ score ≥ 6 at postnatal time point

“Cases” were defined as having an SRQ score of 6 or above, based on a validation study conducted among perinatal women in the study site [22]. The SRQ-20 was specifically designed for the LMIC primary care setting and consists of 20 questions about depressive, anxiety, panic and somatic symptoms present in the preceding month with dichotomous (yes/no) answers [23]. Additionally, the 15-question Patient Health Questionnaire (PHQ-15) was used to assess severity of somatic symptoms. The PHQ-15 asks about 15 different somatic symptoms which have been found to be frequently used as expressions of emotional distress and are rated by respondents from 0 (“not bothered at all”) to 2 (“bothered a lot”) [24]. The 36-item WHO Disability Assessment Schedule (WHODAS II), a generic measure of disability, was used both at the pregnancy and postnatal time points [25]. Data on socioeconomic status (SES) were also collected, including information on household water supply, sanitation, perceived relative wealth compared to others, indebtedness, and experience of hunger in the preceding month. To record a household’s number of assets, an asset inventory was used, including land, a house, a company or business, animals (cows, horses, goats, sheep, etc.), produce or crops, a cooker, a bed, a TV, a radio, jewellery or other valuables, and any other substantial property. All questionnaires were administered in an interview format by lay data collectors who had completed secondary education.

### Fully structured questionnaire data analysis

The measures of somatic symptoms, disability and socio-economic status were analysed descriptively and presented for each individual participant, grouped by SRQ case category.

### Qualitative data collection

Semi-structured interviews were conducted to elicit in-depth information with regard to perinatal mental distress. The interviews took place after the 2-month postnatal questionnaire assessment, with the time period in between ranging from a few days to up to 3 months. All interviews were carried out in Amharic by psychiatrists from Addis Ababa University who were trained in qualitative interviewing techniques. The interviews took place in the woman’s home. Interviewers introduced themselves as researchers interested in learning about women’s experiences after giving birth and the problems that may arise. The qualitative interviews were exploratory and aimed to elicit women’s perspectives on a wide range of topics related to pregnancy, childbirth, and the postnatal period. Domains covered in the interview guide included free-listing of health problems that can be associated with childbirth; an illness narrative including labelling, symptom patterns, severity and timing; practical and emotional support; rituals of child-bearing; and explanatory models. Interviewing was continued until the investigators considered that theoretical saturation had been achieved.

Interviews were audio recorded (with permission), transcribed in Amharic and then translated into English before coding. The translator noted any areas where they felt there was ambiguity, and these were discussed with the researchers.

### Qualitative data analysis

Interview transcripts were analysed using qualitative data analysis software (NVivo 11.4). The first author (JM) and second author (CH) read through all transcripts. JM formulated codes and discussed these with CH and EGJS. Codes were guided by the themes defined in the topic guide, as well as by topics that emerged from the data, using the principles of thematic analysis [26]. JM then identified key themes and explored relationships between them through a process of memoing. JM retrieved data segments corresponding to the main themes, and shared and discussed these with EGJS in relation to the study objectives.

### Triangulation

The sequential explanatory study design allowed for triangulation of the different data types. Integration occured both through *building*, as the fully-structured questionnaire data informed the purposive sampling strategy for the qualitative data collection, and through *merging*, as the two types of data were brought together for analysis and comparison [27]. The qualitative results refined interpretation of the questionnaire data and placed them in appropriate context. Combining the two datasets also allowed for analyses of areas of concordance and discordance between emic and etic perspectives on perinatal mental distress. For example, patterning of quantitative socio-economic status indicators could be linked to explanatory models expressed in qualitative interviews; and prominent symptoms identified in qualitative interviews could be compared with those included in the fully-structured questionnaires.

## Results

We present results in three sections: I: fully-structured questionnaire results, II: qualitative results, and III: integration of data sources.

### I: fully-structured questionnaire results

See Table 3.

**Table 3:** Descriptive summary of quantitative measures stratified by individual participants

SRQ case classification	Qual ID	SRQ-20 score		WHODAS score		PHQ-15 score		No. of assets (AN only)	Self-reported experience of hunger in the past month	
		AN	PN	AN	PN	AN	PN		AN	PN
Non-case throughout	11	3	2	6	19	0	3	3	no	yes
	12	3	4	6	6	1	0	4	no	no
	18	0	0	6	5	2	1	5	yes	no
	20	3	1	3	0	0	2	7	no	no
Case throughout	1	18	20	24	33	3	0	2	yes	yes
	2	14	18	43	75	0	0	3	yes	yes
	6	13	7	19	0	5	1	2	yes	yes
	7	6	15	14	46	0	5	5	yes	yes
	8	19	14	67	61	11	0	5	no	no
	9	8	14	6	10	1	2	4	yes	no
	10	9	9	28	17	3	1	5	yes	yes
	17	15	13	18	11	5	2	2	no	no
	21	7	8	3	17	0	0	5	no	no
Pregnancy case resolving	3	15	0	32	7	3	0	4	yes	no
	4	14	1	46	0	5	2	4	yes	yes
	5	6	3	39	6	4	4	5	no	no
	13	10	4	33	9	0	1	2	yes	no
	15	10	2	9	0	0	0	2	yes	no
New-onset postnatal case	14	3	8	5	0	2	1	6	no	no
	16	2	15	3	4	3	1	5	yes	no
	19	2	18	5	9	0	1	5	no	yes
	22	3	12	0	20	1	10	3	no	yes

*AN* antenatal, *PN* postnatal

Patterns were observed between some of the socio-economic status indicators and SRQ-20 classifications. In general, if a woman scored above the SRQ threshold for caseness, she tended to have reported being hungry in the past month. Similarly, women who were SRQ-20 cases at the antenatal timepoint typically reported a lower number of assets.

### II: qualitative results

#### II.1: Labelling and explanatory models

The majority of women reported that there were no collective ways of labelling perinatal mental distress states. Indeed, most women rejected the idea that they suffered from a disease, instead seeing their distress as a non-pathological reaction to difficult circumstances. One woman explained that her symptoms were simply a result of poverty: “because I have no means to raise my children, nothing to eat or drink, no clothes for my children. Otherwise I have no illness” (*IV04a*). Instead of a distinct local syndrome, women named the symptoms they experienced, which they saw as features of suffering in the context of adversity. The most commonly mentioned symptoms were worry, sadness, anxiety and anger. These emotional states often occurred simultaneously and seemed to reinforce each other. One woman described her feelings as follows: “I feel tense inside and get anxious.… I have the urge to go, to fly away, disappear. I worry a lot. What can I do when I have nothing to feed my children? Sometimes I ask, ‘Shall I die today?’” (*IV04a*). Another woman asked, “Is there anyone who would be happy in this situation? Why wouldn’t I feel sad when I have nothing to provide for my children?” (*IV10*). Feelings were also sometimes characterized using metaphors, such as one woman who said when she walked she felt like “walking on water” and that her thoughts “pulled her heart down” (*IV16*).

The possibility of complications or even death during delivery was a major source of worry for many women. They explained that “you worry whether you live or die” (*IV10*) and this worry might result in “no mental rest day and night” (*IV16*) during the antenatal period. Worrying was often connected to other complaints. For example, women often indicated their sleep was impacted, “because of different thoughts and worries” (*IV14*). Worrying and tension might also result in withdrawal and an inability to function according to social norms (e.g. *IV22*: “I was worrying a lot. I was anxious in my head. I was sitting at home. I was not mixing with people.”). For some, the worrying had reached intolerable levels and they had considered or attempted suicide.

I: Do you often think of killing yourself?

R: Yes, when I have nothing to give to my children I think like that.

I: A-ha.

R: When there is nothing I can do I think like that, and when they shout at me and trouble me. They shout and I have nothing to give them. I worry and I want to die. (*IV04a*)

Many women mentioned that if they could afford it, they could “have a sheep slaughtered” (*IV07*), to provide “blood to drink and good food” (*IV16*). A diet with “butter, milk, meat and gruel”, and “drinking coffee” (*IV03*) were considered as ideal in the postnatal period; the absence of these foods and drinks was considered to lead to distress. Compounding material deprivation, many women were also burdened by an awareness of their *relative* poverty – comparing themselves to others who were slightly better off was often a cause of distress. Not being equal to others, or “the last out of all your neighbours” (*IV07*) was associated with feelings of shame and sadness.

A key factor in the effect poverty had on a woman’s mental wellbeing seemed to be the amount of social support she received. Some women indicated that it was a lack of support that made their situation unbearable (e.g. *IV15*: “It is because I have no one to help me”). If they had had someone to support them, “it would definitely be different and I would not be sick” (*IV11*).

Although the dominant interpretation of experiences that correspond to perinatal distress was as a response to poverty, somatic symptoms were sometimes seen as deriving from a particular illness. One that was frequently mentioned was *berrd*, an illness caused by exposure to cold air. Women were perceived as being vulnerable to *berrd* in the postnatal period because of the “openness” of their bodies after giving birth (*IV18*). Another term specifically used to refer to mental disorder was *likift*, a type of spirit possession. One woman who experienced *likift* said, “It is Satan. It makes people talk, especially at night. For example, sometimes at night I want to go out and run, and when my husband tells me in the morning, I don’t remember it” (*IV17*). Although a majority of women had heard of *likift* and acknowledged such beliefs were widespread, most did not think their symptoms were related to it. Only two women self-identified with *likift* in their qualitative interview.

#### II.2: coping strategies and help seeking

Family support, both emotional and instrumental, was regarded as very important in protecting against excessive worrying. Talking about problems with friends or family was generally perceived to be helpful: even though it might not help her problems go away, “it just helps to discuss the anger and the problem with someone” (*IV06*) and after receiving advice “she will somehow calm down” (*IV01*). Several women mentioned that their relatives were unable to support them because of their own poverty – “you can only give what you have” (*IV01*) – or that they had no living relatives; this was often a source of deep sadness.

There was substantial variation in the amount of support women received from their husbands. For some women, husbands were their main source of support (e.g. *IV03*: “My husband is a very blessed person (…) he helps me a lot”), but most did not think it was appropriate or useful to share their distress with husbands. As one put it, “He is a man. He will only think less of me if I tell him” (*IV18*). For other women, the marital relationship was marked by intimate partner violence (IPV) both during pregnancy and postnatally, including descriptions of beating (*IV21*, *IV22*), hitting (*IV12*, *IV19*) and kicking (*IV17*). Several of the women normalized such experiences as an ordinary aspect of women’s general lack of power (e.g. *IV22*: “What power do I have?”), underlining the lack of support to be obtained from the husband.

Often, it was believed that “only someone close, whom you trust” (*IV02*) should be informed. This likely contributed to the conviction, among many women, that it was not appropriate to seek medical help for their conditions. However, this was not an opinion shared by all respondents, with some women saying that medication (often referred to as ‘tablets’ or ‘injections’) could be effective. “If you have the means, you can get a tablet” (*IV08*).

### III: integration of data sources

We address here first the areas of discordance and then areas of concordance between the fully-structured questionnaire and qualitative results.

The most striking discordance between the two types of data was the case of one woman who had obtained an SRQ score of 0 both at pregnancy and postnatal time point, but who described very severe symptoms in the qualitative interview, including suicidal ideation. Furthermore, there was no clear relationship between reports of improvement in qualitative interviews, and change in scores on SRQ/WHODAS between the antenatal and postnatal period. Nor was there a clear relationship between levels of support as indicated in qualitative interviews, and severity of distress as measured by SRQ score, WHODAS, or suicidality. There was also significant discrepancy between reports of suicidality as they emerged in the interviews and the postnatal SRQ-20 item on suicidality (item 17: *Has the thought of ending your life been on your mind?*). Six of the women who reported suicidal thoughts or feelings in their qualitative interview did not endorse the suicidality item in the postnatal SRQ-20. One of these women had even attempted suicide after giving birth (*IV11*).

In other respects, the qualitative data and fully-structured questionnaire data complemented each other. The frequent mention of somatic symptoms (e.g. physical weakness) in qualitative descriptions of illness was reflected in the WHODAS and SRQ-20 scores for individual women. Furthermore, the emphasis on poverty as a cause of perinatal mental distress in interviews is consistent with the patterning of SRQ caseness with reports of hunger before and after birth. Respondents stressed that the nutritional status of women and infants played a key role in perinatal mental distress. Physical health was considered to be closely related to nutrition: “If you have [enough food], they say, you won’t feel dizzy” (*IV07*). The most plausible explanations for expressing emotional distress in the perinatal period in this setting therefore appeared to be a combination of inadequate food, isolation, and hopelessness caused by poverty.

## Discussion

The results of this study demonstrate that perinatal mental distress states are recognized in this rural setting in Ethiopia, but the ways in which they are experienced do not correspond neatly to Western psychiatric categories. The women interviewed for this study seldom employed the concept of depression, and labelling was instead more closely linked to symptoms. Study respondents typically saw perinatal somatic and emotional symptoms as direct results of hardship, as opposed to signs of a psychiatric condition. The explanations for perinatal mental distress provided by women in this study in Ethiopia related mostly to external factors, as opposed to the biological or genetic factors that are commonly mentioned in a number of European settings [28]. The widespread perception that distress in the perinatal period was primarily a reaction to difficult circumstances means that the use of the Western psychiatric label is unlikely to be acceptable for many of these women.

The absence of a clear relationship between reports of improvement in qualitative interviews and change in scores on SRQ/WHODAS in this study highlights the limitations of fully-structured questionnaire measures. The instance of a clear false negative in this study – a woman who scored 0 on the SRQ-20 both pre- and postnatally but attested to severe symptoms in her in-depth interview – underlines the importance of deepening our understanding of why endorsement of symptoms sometimes differs between the use of screening tools and in-depth interviews.

There are various factors which might hinder disclosure of depressive symptoms in structured questionnaires like the SRQ-20. For some women, there was a time gap of several weeks between the fully-structured questionnaire and qualitative assessments which may also have contributed to discordance, although women were asked about the symptoms they had endorsed previously. In HIC settings, it has been reported that women’s responses to screening might be affected by a desire to handle mental health problems on their own; the emotional cost of retelling; preferring to discuss feelings with significant others; and fear of not having a choice in treatment decisions [29, 30]. In a study that used the SRQ-20 in rural Western India, the authors suggested that women might be less willing to disclose psychological symptoms due to the stigma and social disadvantage associated with mental illness in this setting [31]. Stigma is also likely to be a barrier to disclosure in the Ethiopian context [32, 33]. In addition, the impersonal nature of the questions in the SRQ-20; suboptimal understanding of fully structured questions; and language barriers due to Amharic being the second language for many of the women are likely to play a role. Limited knowledge of mental health disorders, or low ‘mental health literacy’, might mean that it is easier for women to disclose their symptoms in the context of a qualitative interview than within the structured format of a screening tool.

It is also possible that cultural manifestations of mental distress may not be adequately captured by the SRQ-20. In a 2015 validation of SRQ-20 in primary care facilities in small towns in Ethiopia, 50% of gold standard cases of depression expressed ‘irritability’ rather than the core depressive symptoms from international criteria of persisent low mood or loss of enjoyment of pleasurable activities [34]. This suggests that screening for perinatal mental distress in this setting may need to attend more to ‘irritability’, a symptom not included in the The Diagnostic and Statistical Manual of Mental Disorders (DSM) for adult depression [35].

The most common explanation for perinatal distress provided by women in this study, as rooted in material deprivation, is consistent with the results of other qualitative [12, 16, 17, 36–38] and quantitative studies [39, 40]. For women struggling due to poverty, the experiences of pregnancy, childbirth and care for an infant can add additional layers of stress, reducing their ability to cope with their circumstances [16, 17, 41]. Causality may run both ways, with poverty and mental ill-health during the perinatal period mutually reinforcing each other. Poverty increases a woman’s risk of malnutrition and obstetric complications and reduces her access to social capital, while mental distress may lead to reduced productivity and social drift, thereby aggravating poverty [39]. The apparent impossibility of real transformation in life may contribute to what Duflo has described as a ‘hopelessness-based poverty trap’, whereby the poor do not have the energy or mental space to do anything more than just scrape by [42]. The importance of relative, rather than absolute, poverty for the risk of depression in women reported in this study has also been observed in a national survey in the United States [43].

Physical health issues were also important factors in explanatory models of perinatal mental distress. Poor physical health – whether caused by malnutrition, illness, or childbirth – was seen as a significant contributing factor to perinatal mental distress, echoing findings from studies in other low-resource settings [14, 36, 44–46]. Symptoms of physical ill-health may be both cause and manifestation of perinatal mental distress, as previously reported in the literature. For example, Indian women have been reported to use vaginal discharge as an idiom of distress, reflecting broader issues of social stress [47]. In previous analyses from the same cohort of perinatal women in Ethiopia, complaints of fatigue were strongly associated with SRQ-20 scores and psychosocial stressors, but not with haemoglobin levels or undernutrition [48], further highlighting the importance of physical symptoms in emotional distress.

Consistent with other qualitative findings, the dangers of pregnancy and birth were identified as prominent sources of worry during the prenatal period [14, 16, 45] and the risks faced by the baby as a major cause of concern postnatally [14, 36]. These concerns are experienced by mothers in HICs too [28] but they are surely reinforced in settings with higher rates of maternal and infant mortality [49], and are likely exacerbated by the clustering of maternal and infant deaths among families of lower socioeconomic status within LMICs [50].

Finally, supernatural factors (e.g. *likift*) played a role in a few women’s explanatory models, resonating with findings from other sub-Saharan African settings. In Tanzania, for example, possession by shades (*mizimu*), devils (*shetani*) or spirits (*majini*) were mentioned as causes of mental health problems for perinatal women [44]. As supernatural explanations are often related to interpersonal conflict, these causal explanations may have a communicative function by providing a culturally sanctioned way for women to ask for help from the community, as described in Malawi [14].

Although several women reported experiencing IPV, this was not typically linked to explanatory models of perinatal mental distress. Normalisation of IPV should be seen in the context of its high prevalence in Ethiopia, with more than 1 in 4 women experiencing IPV during pregnancy [51]. The absence of IPV in explanatory models is at odds with quantitative data from the Butajira demographic surveillance site reporting an association between IPV and depressive episodes [52]. Women’s normalising or accepting attitudes toward IPV are likely linked to their low empowerment status [53].

In terms of coping strategies and help-seeking, women in this study indicated that talking about their problems with others could be useful. While some women shared their worries with their husbands, others preferred talking to family and friends. This corresponds to findings from a quantitative study of coping strategies of women with postpartum depression symptoms in a neighbouring district in Ethiopia, which reported that the coping strategy of ‘getting comfort and understanding from someone’ was frequently used [54]. The most frequently employed coping strategies identified in that study were religious coping strategies (praying; meditating; finding comfort in religious or spiritual beliefs), which were not mentioned by any of the participants in this study [54]. Some women in this study did not think it was valuable to inform others of their distress at all. Concealing troubles from others or ‘taking personal responsibility’ [55] has been described in other low-resource settings as well, sometimes to protect the family reputation [56]. Discouragement of active help-seeking from an outsider or professional was often related to shame and stigma, but also to a perceived lack of treatment options. The notion that ‘doctors don’t help with sadness’ expressed by some women in this study was echoed by women in Goa, India [57] and Dhaka, Bangladesh [56]. However, there was variation in attitudes towards medical help seeking in this sample, because some women did feel their problems were amenable to biomedical solution, and that medicines might be able to help them. In a community based cross-sectional survey conducted in southern Ethiopia, 49.9% of the 276 (71.6%) women with postpartum depression who said their illness needed treatment favoured modern medicine as their chosen type of treatment [58]. This suggests potential acceptability of health facility-based treatment interventions to address problematic perinatal mental distress in this setting.

### Study limitations

Ethiopia’s diverse population and the use of a purposive sampling technique limits the generalisability of our findings to all Ethiopian women, although the main themes identified in this study reflect issues that are pertinent in rural areas across the country. The translation of transcripts into English may have resulted in loss of some of the specific meaning of women’s experiences, despite efforts to address areas of ambiguity by the translator. Furthermore, social desirability bias may have led some women to conceal certain issues or thoughts in the qualitative interview. The use of (predominantly male) psychiatrists as interviewers may also have affected women’s responses, although most women did not actually endorse psychiatric categories. Another potential limitation of the study is that the data are 14 years old, and both maternal and infant mortality have declined substantially since then [59, 60]. Nonetheless, levels of mortality remain unacceptably high and an important back-drop to the lives of women [61]. The area remains predominantly rural, with high levels of poverty, low access to quality reproductive healthcare, and low coverage of electricity and safe water sources [61]. Furthermore, qualitative studies of perinatal women from better-resources settings in other LMICs [12, 36, 37] have reported similar findings, indicating that these are not transient, circumscribed issues. Finally, the small sample size and small numbers of respondents in each of the subcategories can be seen as a limitation. However, saturation was achieved through the course of the interviews.

## Conclusions

In conclusion, most women in this Ethiopian setting recognised the existence of perinatal mental distress states, but typically considered such distress as a non-pathological reaction to difficult circumstances. This study’s findings further highlight the importance of the wider social context within which mental health problems are situated, including their inseparability from issues of gender, physical health and poverty. This implies that public health prevention strategies, assessments and interventions for perinatal distress should be developed from the bottom-up, taking account of the local context and explanatory frameworks.

## Data Availability

The datasets analysed during the current study are not publicly available due to the risk to the individual privacy of the study participants, but are available from the corresponding author on reasonable request.

## Abbreviations

LMICs: Low and Middle-Income Countries
HICs: High Income Countries
P-MaMiE: Perinatal Maternal Mental disorder in Ethiopia
CMD: common mental disorder
SRQ-20: Self-Reporting Questionnaire 20-item
PHQ-15: Patient Health Questionnaire
SES: Socioeconomic Status
WHODAS: WHO Disability Assessment Schedule
IV: Interview
IPV: Intimate Partner Violence
